# Proteome analysis of *Phytomonas serpens*, a phytoparasite of medical interest

**DOI:** 10.1371/journal.pone.0204818

**Published:** 2018-10-10

**Authors:** Agenor de Castro Moreira dos Santos Júnior, Carlos André Ornelas Ricart, Arthur Henriques Pontes, Wagner Fontes, Agnelo Rodrigues de Souza, Mariana Souza Castro, Marcelo Valle de Sousa, Beatriz Dolabela de Lima

**Affiliations:** 1 Laboratory Protein Chemistry and Biochemistry, Department of Cell Biology, University of Brasília, Brasília, Federal District, Brazil; 2 Laboratory of Gene Biology, Department of Cell Biology, University of Brasília, Brasília, Federal District, Brazil; Tulane University, UNITED STATES

## Abstract

The protozoan *Phytomonas serpens* (class Kinetoplastea) is an important phytoparasite that has gained medical importance due to its similarities to *Trypanosoma cruzi*, the etiological agent of Chagas disease. The present work describes the first proteome analysis of *P*. *serpens*. The parasite was separated into cytosolic and high density organelle fractions, which, together with total cell extract, were subjected to LC-MS/MS analyses. Protein identification was conducted using a comprehensive database composed of genome sequences of other related kinetoplastids. A total of 1,540 protein groups were identified among the three sample fractions. Sequences from *Phytomonas* sp. in the database allowed the highest number of identifications, with *T*. *cruzi* and *T*. *brucei* the human pathogens providing the greatest contribution to the identifications. Based on the proteomics data obtained, we proposed a central metabolic map of *P*. *serpens*, which includes all enzymes of the citric acid cycle. Data also revealed a new range of proteins possibly responsible for immunological cross-reactivity between *P*. *serpens* and *T*. *cruzi*.

## Introduction

*Phytomonas serpens* is a flagellated protozoan (class Kinetoplastea, order Trypanosomatida [1]) of considerable agricultural importance as a phytoparasite of tomato crops. This protozoan, which is transmitted by the vector *Phthia picta*, retains a promastigote form during its entire life cycle. To date, little is known about *P*. *serpens* in terms of its’ biology, life cycle, or how the species has adapted to life inside plants [2–4].

The members of the class Kinetoplastea are peculiar organisms that differ from most other eukaryotes in a number of biological features. Notable differences include the presence of organelles such as glycosomes and the existence of a single mitochondrion bearing a complex array of DNA called kinetoplast [5]. Trypanosomatids also possess peculiar characteristics during cell division, including closed mitosis, an absence of chromosome condensation and replication of DNA at the periphery of the nucleus [6–8]. Furthermore, *Phytomonas* species lack most of the known hemeproteins and do not require heme groups for the transport of electrons along the respiratory chain and for other important metabolic processes [9].

A number of previous studies have applied proteomic strategies to better understand the peculiar biology of members of the kinetoplastids, with reports for *Trypanosoma cruzi*, *Trypanosoma brucei* and *Leishmania* [10–18]. Currently, liquid chromatography-tandem mass spectrometry (LC-MS/MS) has become the gold standard method to analyze proteins from complex biological samples, since it allows the identification and the quantification of thousands of proteins in a single experiment [19]. Moreover, data from LC-MS/MS experiments can have a positive impact on poorly annotated genomes by contributing to the curation process [20]. Such a strategy has been applied to *Leishmania donovani* [21].

*P*. *serpens* has gained importance in medical research due to its similarities to *Trypanosoma cruzi*, the etiological agent of Chagas disease. Previous studies have shown that chagasic patients display antibodies which are able to recognize *P*. *serpens* antigens [22–24]. Furthermore, intraperitoneal and oral route inoculation of mice with *P*. *serpens* was reported to promote some protection against *T*. *cruzi* infection. Consequently, authors claimed that oral immunization with *P*. *serpens* might constitute an alternative vaccination approach to *T*. *cruzi* infection [24].

In the present work, we carried out a proteomic analysis of *P*. *serpens* via LC-MS/MS and employed databank sequences from all kinetoplastids available in UNIPROT for protein identification. We were able to identify 4,387 proteins with at least one unique peptide. Moreover, *P*. *serpens* proteome characterization has enabled the generation of useful data which may provide support to research on the biology and the mechanisms of pathogenicity in kinetoplastids.

## Material and methods

### Cell culture and fractionation

*P*. *serpens* (strain 9T) was grown in brain heart infusion broth (BHI) medium (Acumedia, Lansing, Michigan) at 27 °C. Parasite cells (5 x 10^8^) collected in logarithmic growth phase were fractionated using a protocol based on a previously described report [25]. Parasites were centrifuged for 10 min at 5,000 *g* then washed three times in phosphate buffered saline (PBS). The pellet was resuspended in eight volumes of hypotonic buffer TENM2 (10 mM Tris-HCl pH 7.4, 10 mM NaCl, 1 mM MgCl_2_, 1 mM MnCl_2_, 5 mM β-mercaptoethanol) and cell turgidity was confirmed by optical microscopy. Nonidet P40 (0.5% (v/v) final concentration) and protease inhibitors (cOmplete Mini, Roche, Meylan, France) were added to the cells, which were then lysed using a Dounce homogenizer. Cell lysis was followed by optical microscopy. Osmolarity of the lysate was reestablished by adding 12.5% (v/v) of 2 M sucrose (0.25 M final sucrose concentration). The sample was then transferred to a conical centrifuge tube containing 5 ml of 0.58 M sucrose in TENM2 and centrifuged at 2,000 *g* for 10 min. The top layer contained the cytosol (CYT), with the pellet containing the high-density organelles (HDO).

### Protein digestion

CYT and HDO fractions, as well as total *P*. *serpens* cells (TOTAL), were trypsin digested using the filter-aided sample preparation (FASP) protocol adapted from [26]. Briefly, each sample was solubilized in LB1 lysis buffer (4% (w/v) SDS, 0.02 M TEAB, 0.1 M DTT, pH 7.9) and heated at 90 °C for 10 min. Samples were then submitted to sonication using a GE50T ultrasonic processor (Cole–Parmer, Chicago, USA). For that, 3 x 10 s cycles at 40% maximal power were employed. Samples were then centrifuged at 16,000 *g* for 15 min and proteins present in the supernatant were quantified using Qubit Protein Assay (Thermo Fisher Scientific, Waltham, USA).

Aliquots from each sample containing 30 μg of protein were diluted in 200 μl of UA (8 M Urea, 0.02 M TEAB, pH 8.5) in a 30 kDa filter unit (Sartorius, Goettingen, Germany) and centrifuged at 14,000 *g* for 15 min. The filter unit was then washed with 200 μl of UA, centrifuged at 14,000 *g* for 15 min and the flow-through discarded. Subsequently, 100 μl of IAA (0.05 M iodoacetamide in UA) solution was added and the system was incubated in a Thermomixer mod. 22331 (Ependorff, Hamburg, Germany) at 600 rpm for 1 min followed by 20 min at 21°C without mixing. Filter units were then centrifuged at 14,000 *g* for 10 min. IAA excess was removed with 100 μl of UA and 14,000 *g* centrifugation for 15 min. A volume of 100 μl of 0.02 M TEAB pH 7.9 was added to the filter unit and centrifuged at 14,000 *g* for 10 min. This step was repeated one more time, followed by the addition of 90 μl of 0.02 M TEAB, pH 7.9, containing trypsin (1:100 enzyme: protein ratio). The filter units were mixed at 600 rpm in the Thermomixer for 1 min and incubated in a wet chamber at 37°C for 18 h. The resulting tryptic peptides were collected by addition of 210 μl of water, followed by centrifugation as described above. The sample was acidified with 7.5 μl of 20% TFA and desalted using C18 Ultra-Micro Spin columns (Harvard Apparatus, Holliston, MA, USA). The resulting samples were dried in a vacuum centrifuge.

### 1.1 LC/MS-MS analyses

Protein digests of each sample were loaded (3 μg of total peptide) onto a Reprosil-Pur 120 C18-AQ in-house packed trap column (5 μm particle size, 5.0 cm length, 100 μm inner diameter, 360 μm outer diameter) using an UltiMate 3000 Nano LC (Dionex, Amsterdam, The Netherlands). The trap column was washed for 5 min with solvent A (0.1% (v/v) formic acid, 2% (v/v) acetonitrile). Peptides were eluted onto a C18 Reprosil-Pur 120 C18-AQ (3 μm particle size, 23 cm length, 75 μm inner diameter, 360 μm outer diameter) in-house packed analytical column at a flow rate of 230 nL.min−1. The gradient comprised 10–35% of solvent B (0.1% (v/v) formic acid, 95% (v/v) acetonitrile) for 155 min. Peptides were electrosprayed into an LTQ-Orbitrap Elite mass spectrometer (Thermo Fischer Scientific, Bremen, Germany) via a nanospray probe (Thermo Scientific, Germany) with a spray voltage of 3.02 kV and transfer capillary temperature set to 275 °C. The mass spectrometer was operated in Data Dependent Acquisition (DDA) mode using Xcalibur 2.2 software (Thermo Scientific). The acquisition cycle consisted of a survey scan from 300–1650 m/z at 120,000 resolution (full width at half maximum) at m/z 400 using one microscan in the Orbitrap, followed by fragmentation of the 15 most intense multiply charged precursors using higher energy collision induced dissociation (HCD) under normalized collision energy of 35. Fragmentation was also performed by collision induced dissociation (CID) fragmentation of the 20 most intense multiply charged precursors in new LC-MS/MS runs. The ion selection threshold for MS/MS was set to 3,000 counts using a precursor isolation window of 2 amu, while dynamic exclusion was set at 30 s.

### Bioinformatics

Data files were analyzed using the software PEAKS (Version 7; Bioinformatics Solutions Inc., Waterloo, Ontario, Canada). A local database was employed comprising available Kinetoplastea parasite protein sequences for more than 200 different species (http://www.uniprot.org; release oct_2015) including two *Phytomonas* spp. (HART1 from group H and EM1 from group D), *Trypanosoma cruzi* (CL Brener), *Trypanosoma brucei* and *Leishmania infantum* (S1 Database). Criteria for protein identification included the detection of at least one unique tryptic peptide and false-positive discovery rate (FDR) of less than 1%. Tolerance filters of 0.5 Da for precursor ions and 10 ppm for fragment ions were employed. Carbamidomethylation of cysteine was considered a fixed modification, while acetylation of the N-terminal and methionine oxidation were set as variable modifications.

Gene ontology analyses were performed in Blast2GO (version 4.1.9) and DAVID bioinformatics resources 6.7 (http://david.abcc.ncifcrf.gov/) using the software default parameters.

## Results and discussion

Most proteomic studies concerning kinetoplastids have been supported by the availability of complete and annotated genome sequences of human pathogens such as *T*. *cruzi*, *T*. *brucei*, *Leishmania* and several other organisms [13,27–31]. In contrast, the present study was performed to analyse the proteome of *P*. *serpens*, a phytopathogenic kinetoplastid. Although the *P*. *serpens* genome was previously sequenced in order to enable identification of heme-containing proteins in this parasite [9], the genome sequence unfortunately did not provide annotation of protein coding genes, which is crucial for proteomic analysis. Given this, we compiled a comprehensive reference database composed of genome sequences for closely related species, from *T*. *cruzi*, *T*. *brucei*, *L*. *infantum* and a compilation of other species belonging to the Kinetoplastea class (S1 Database).

**Fig 1:**
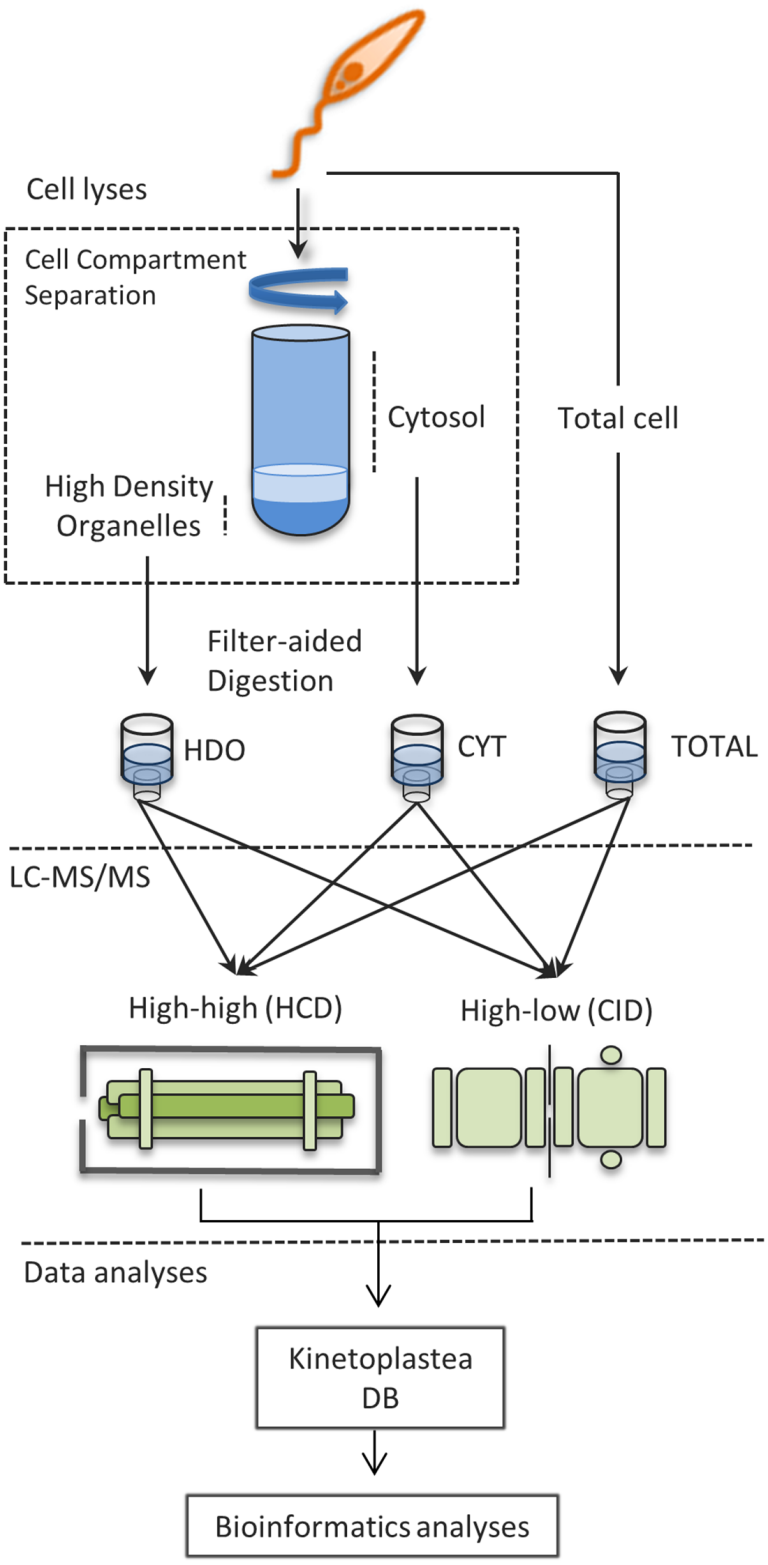
Cell fractionation and proteomic strategy analyses.

*P*. *serpens* cells were firstly fractionated into a cytosolic (CYT) and a high-density organelle fraction (HDO). This fractionation into two different subproteomes was conducted as a strategy to decrease sample complexity for LC-MS/MS analysis, a step usually performed to increase the likelihood of identifying proteins sometimes underrepresented in global proteomic assays, as previously demonstrated [32]. In addition, total cell extracts (TOTAL) were also subjected to proteomic analyses. The overall strategy outlined in Fig 1 resulted in the identification of 2,949 proteins in CYT; 2,976 in HDO; and 3,807 in TOTAL (S1 Table, MS/MS raw data files are available at PeptideAtlas, dataset identifier PASS01214). These proteins were distributed in 1,540 different protein groups. Detailed information about the peptides identified in each experimental condition is available in S2 Table.

**Fig 2:**
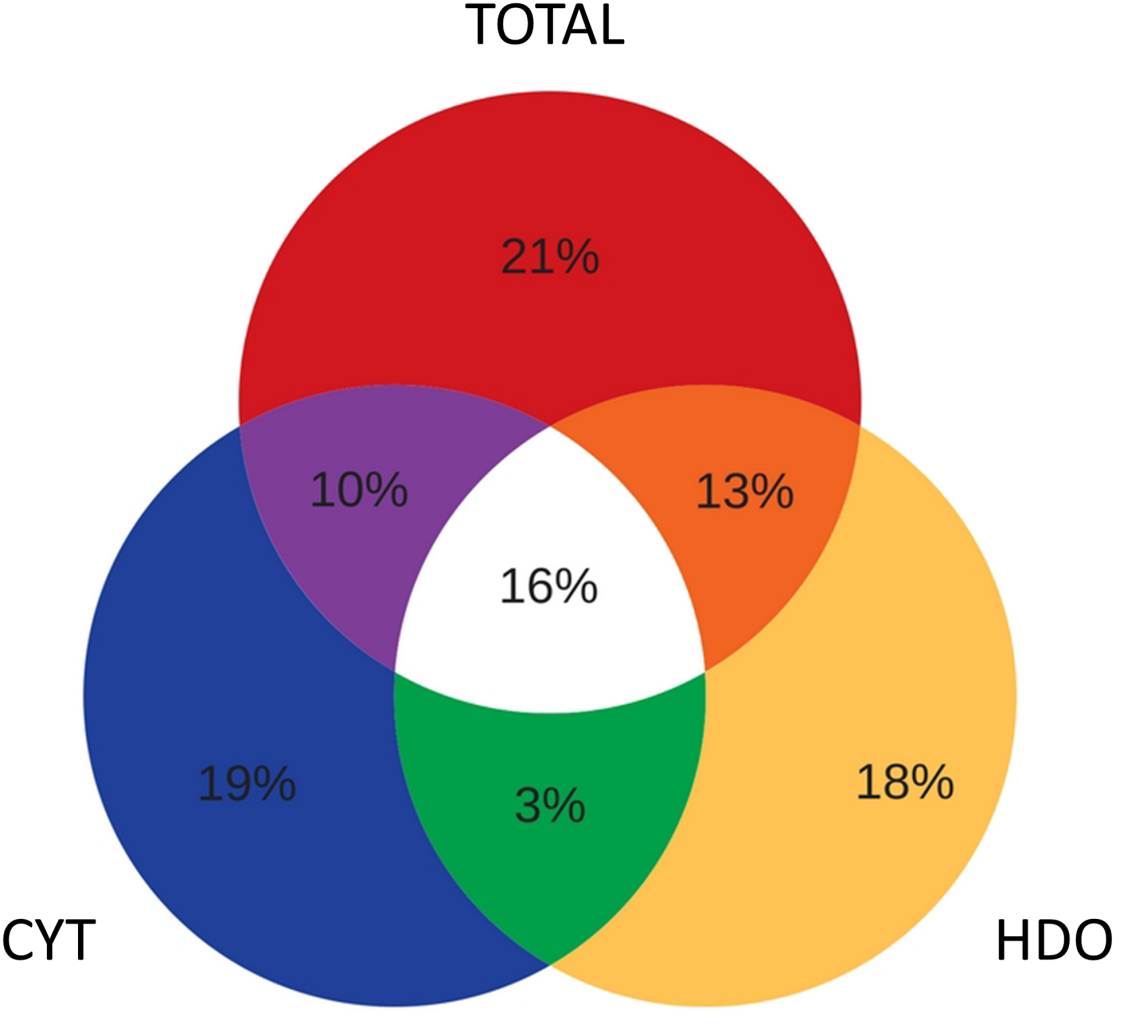
Venn diagram showing the distribution of identified proteins in TOTAL (total cell proteome), CYT (cytosol) and HDO (high density organelles) fractions.

Fig 2 illustrates the distribution of the identified proteins among CYT, HDO and TOTAL. The Venn diagram shows that around 18% of the proteins were exclusively identified within the CYT fraction, 18% in HDO and 20% in TOTAL. Interestingly, 58% of the proteins were identified in just one of the fractions (CYT, HDO or TOTAL), showing that the approach used here succeeded in providing a larger proteome data set. In addition, we observed that the average sequence coverage of proteins identified in CYT and HDO (19.2% and 18.5% respectively) was higher than in the TOTAL fraction (16.8%). This highlights that the fractionation improved the coverage of the identifications.

**Fig 3:**
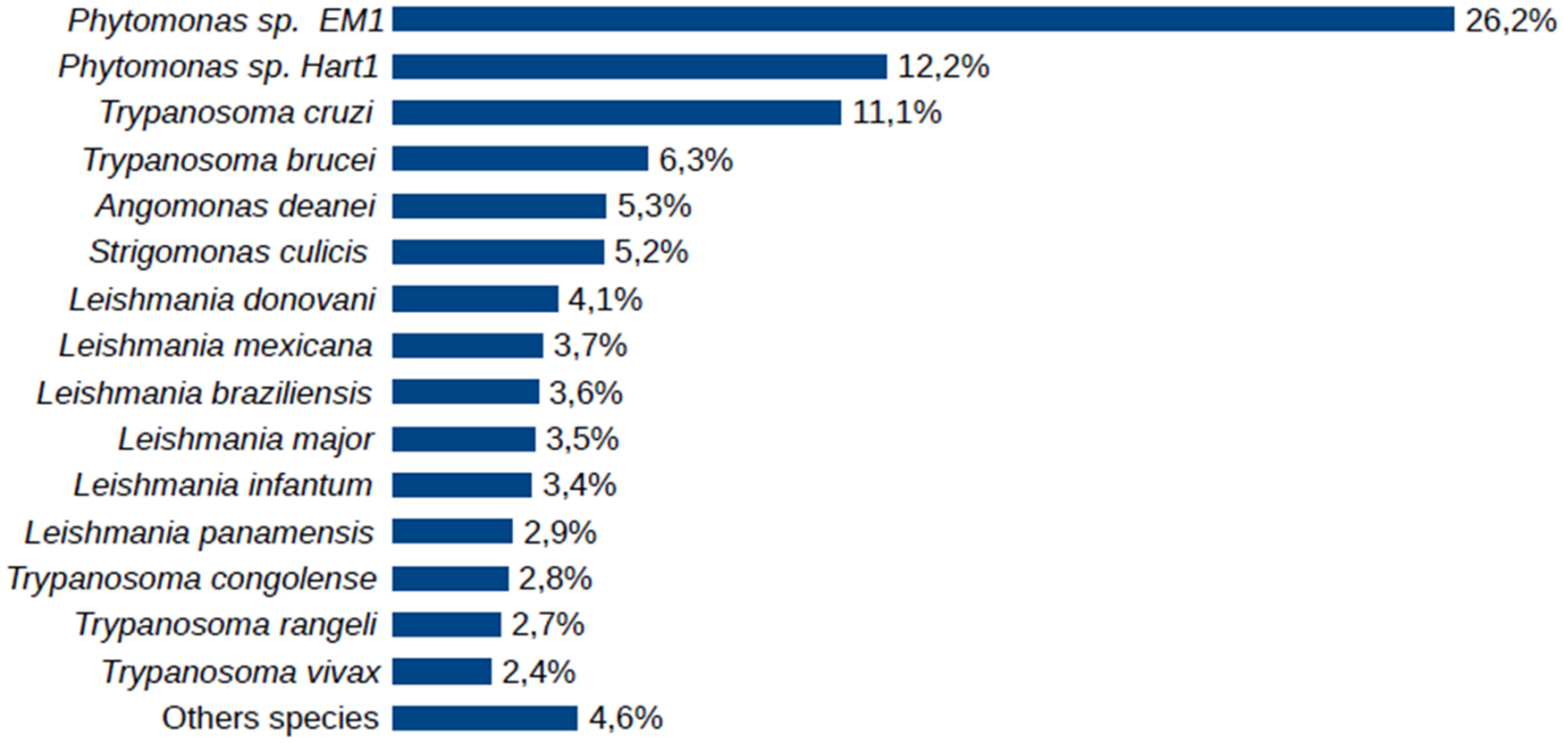
Distribution of proteins identified in the *P*. *serpens* proteome in terms of the sequence databank species.

Among the organisms used in construction of the local database, *Phytomonas* sp EM1 and HART1 provided more protein hits in the identification process than the other species (Fig 3). This result was expected since they agree with previous phylogenetic studies based on HSP90 sequence homology [30]. However, although this phylogenetic study showed *Phytomonas serpens* closer to Leishmanias [30], *T*. *cruzi* and *T*. *brucei* were the human pathogens that provided more protein matches.

**Fig 4:**
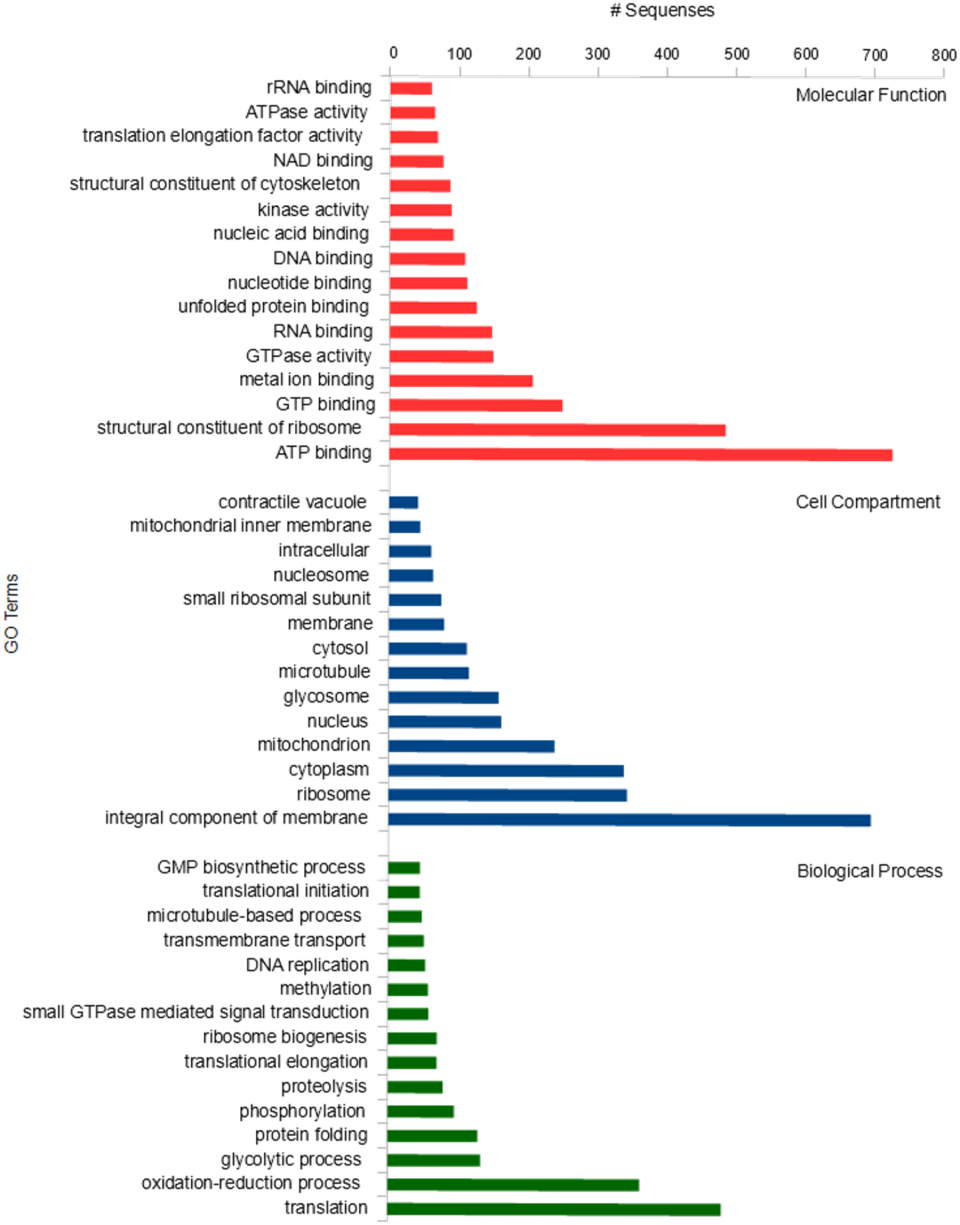
GO terms of proteins found in the *P*. *serpens* proteome relateing to molecular function, cellular component and biological activity.

We performed functional annotation of all the identified proteins using Blast2GO, with 73% of proteins grouped in GO terms related to molecular function, biological process or cellular component (Fig 4). The most abundant molecular function terms were those related to binding activities, including ATP, GTP, metal ion, RNA, unfolded protein, nucleotide, DNA, nuclei acid and NAD binding proteins. Overall, Blast2GO classified 148 proteins under RNA binding, which can be considered very relevant for kinetoplastids [33], as gene expression is post-transcriptionally regulated in these organisms [34].

Gene ontology analysis classified 697 proteins as integral components of the membrane, this being the cell compartment with highest number of hits. In part, the identification of a large number of those proteins might be explained by the method of choice used for protein digestion. Previous studies have shown that filter-aided sample preparation (FASP) enables an increase in the identification of membrane proteins when compared with other digestion protocols [26] Moreover, gene ontology analysis also revealed proteins associated with other cellular structures such as ribosome, mitochondrion, nucleus and glycosome. For instance, 159 proteins were assigned to glycosome–a spherical cellular structure found in trypanosomatids–, which is a special type of peroxisome and contains the main enzymes of the glycolytic pathway [35].

The investigation of *P*. *serpens* metabolism is vital to understand its pathogenicity to plants and for comparative analysis to other trypanosomatid flagellates that share evolutionary relationships with this phytopathogen. In general, trypanosomatids produce and secrete still-reduced carbon compounds from glucose catabolism (e.g. pyruvate, ethanol, acetate, alanine) even under aerobic conditions, instead of oxidizing glucose completely to CO_2_ and water [36].

**Fig 5:**
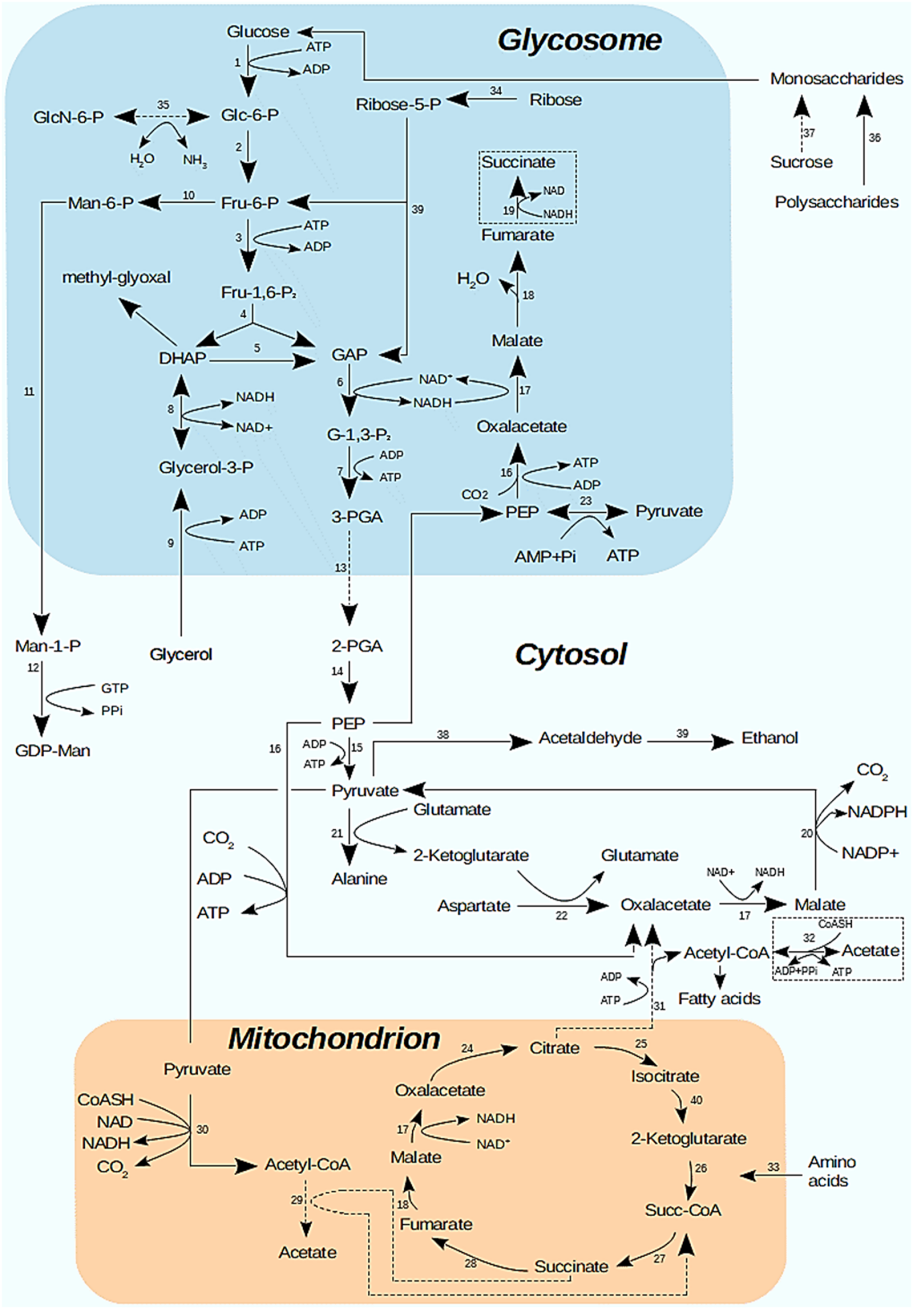
Proposed *P*. *serpens* metabolic map based on the present proteomic data as compared to those of *Phytomonas* sp. EM1 and HART1 [30]. Dashed boxes show enzymes found in the present *P*. *serpens* proteome analysis but not in *Phytomonas* sp EM1 and HART1 genome [30]. Dashed arrows correspond to enzymes found in *Phytomonas* sp EM1 and HART1 genome but not in the present work. Solid arrows represent enzymes found in *P*. *serpens* proteome and in *Phytomonas* sp EM1 and HART1 genome. Enzymes: 1, hexokinase; 2, glucose-6-phosphate isomerase; 3, 6-phosphofructokinase; 4, fructose-bisphosphate aldolase; 5, triosephosphate isomerase; 6, glyceraldehyde-3-phosphate dehydrogenase; 7, phosphoglycerate kinase; 8, glycerol-3-phosphate dehydrogenase; 9, glycerol kinase; 10, mannose-6-phosphate isomerase; 11, phosphomannomutase; 12, mannose-1-phosphate guanyltransferase, 13, phosphoglycerate mutase; 14, enolase; 15, pyruvate kinase; 16, phosphoenolpyruvate carboxykinase; 17, malate dehydrogenase; 18, fumarate hydratase; 19, NADH-dependent fumarate reductase; 20, malic enzyme; 21, alanine aminotransferase; 22, aspartate aminotransferase; 23, pyruvate phosphate dikinase; 24, citrate synthase; 25, isocitrate dehydrogenase; 26, 2-oxoglutarate dehydrogenase; 27, succinate-CoA ligase; 28, succinate dehydrogenase; 29, acetate: succinate CoA transferase, 30, pyruvate dehydrogenase; 31, citrate lyase; 32, acetyl-CoA synthetase; 33, amino acid oxidation pathway; 34, ribokinase; 35, glucosamine-6-phosphate deaminase; 36, glucoamylase; 37, invertase; 38, pyruvate descarboxylase, 39, alcohol dehydrogenase, 40. isocitrate dehydrogenase.

Many enzymes of metabolic pathways located in the glycosome, in the mitochondrion and in the cytosol, including glycolysis and citric acid cycle, were observed in the *P*. *serpens* proteome. These proteins allowed us to propose a central metabolic map (Fig 5) for *P*. *serpens* based on the core metabolism pathways previously published for two *Phytomonas* sp. (EM1 and HART1) and *Leishmania major* [30].

Whilst most enzymes described in the core metabolic pathway of *Phytomonas* sp. were also found in the *P*. *serpens* proteome, we also identified a NADH-dependent fumarate reductase and an acetyl-CoA syntetase, which have not been observed in the *Phytomonas* sp. (EM1 and HART1) genomes. The enzymes phosphoglycerate mutase, citrate lyase, glucosamine-6-phosphate deaminase and acetate: succinate CoA transferase deaminase could not be identified in the present work. However, we managed to find sequences which probably code for homologs of phosphoglycerate mutase (85% identity), citrate lyase (56% identity), glucosamine-6-phosphate deaminase (73% identity) but not for acetate: succinate CoA transferase in the non-annotated *P*. *serpens* genome sequence [9] (S1 Fig). Interestingly, enzymes found in the EM1 genome but absent from HART1 (i.e. malic enzyme and phosphomannomutase) were identified in our study. This is in agreement with positioning in a phylogenetic tree of trypanosomatids based on HSP90 sequence homology that places *P*. *serpens* closer to EM1 than to HART1 [30].

Chaumont and collaborators [37] demonstrated, using enzymatic and NMR measurements, that the major end-products of glucose catabolism of *Phytomonas* sp. isolated from *Euphorbia characias* under aerobic conditions were acetate, ethanol and carbon dioxide. The pathways related to the production of ethanol and carbon dioxide were identified in this work. Although the enzyme acetate: succinate CoA transferase, that converts acetyl-CoA to acetate, was not found here, acetate could be produced from acetyl CoA by acetyl-CoA synthetase. In this sense, all enzymes of glycolysis, apart from phosphoglycerate mutase, were identified (Fig 5). Also, our data revealed pyruvate/indolepyruvate decarboxylase, a key enzyme in alcoholic fermentation previously characterized in *P*. *serpens* [38], and alcohol dehydrogenase. These results ratify that ethanol is produced in aerobic conditions, and this metabolic route is an alternative and necessary route to reoxidize part of the NADH produced in the highly demanding glycolytic pathway [38]. The absence of invertases in the protein lists suggests that *P*. *serpens*, as with *Phytomonas* sp. EM1 and HART1 [30], does not have the capacity to convert sucrose to glucose and fructose.

*Phytomonas* isolated from *Euphorbia characias* were shown to contain high activities of enzymes involved in the hydrolysis of polysaccharides into monosaccharides [39]. However, no amylases, amylomaltases, invertases or carboxymethyl cellulases were identified in the present work. Porcel and collaborators [30] investigated *Phytomomas* sp. putative secreted proteins (containing a secretion signal peptide, no transmembrane domains and no GPI anchors) involved in carbohydrate degradation. They found a sequence containing a glycoside hydrolase family 31 domain in both isolates (HART1 and EM1) and a secreted beta-fructofuranosidase in HART1 only. However, expression data did not show translation of any proteins likely to be involved in plant cell degradation. The authors pointed out that *Phytomonas* does not need to degrade cell walls to penetrate the host, since the parasite is directly injected in the plant phloem by the insect vector.

The citric acid cycle in *Phytomonas* has been described as nonfunctional, as the mitochondria are not capable of oxidizing 2-ketoglutarate, succinate and proline [39]. However, a recent study showed that 2-ketoglutarate dehydrogenase, succinate dehydrogenase and proline oxidation pathway enzymes are present in the *Phytomonas* sp (EM1 and HART1 isolates) genome [30]. Surprisingly, all the enzymes belonging to the citric acid cycle have been found in the *P*. *serpens* proteome, raising once again the question as to whether the mitochondrion is metabolically inactive, as it was once proposed for *Phytomonas* sp. [37], or if it is active, but only under certain conditions.

*Phytomonas serpens* is an eukaryote able to survive in the absence of heme-proteins, as it does not require heme for electron transport in the respiratory chain, protection against oxidative stress or desaturation of fatty acids [9]. As expected, cytochromes of the electron transport chain were not found in this proteome data set. However, the lanosterol 14-alpha-demethylase (ortholog of *T*. *brucei*), a heme-protein involved in the synthesis of sterols, such as cholesterol and ergosterol [40] is available in the proteome. The presence of lanosterol 14-alpha-demethylase in *P*. *serpens* had been previously described [9].

The absence of cytochrome-mediated respiration in *P*. *serpens* results in a limited mitochondrial role in energy metabolism of this phytoparasite [41]. Still, *P*. *serpens* has a short mitochondrial electron transport chain based on an alternative oxidase (salicylhydroxamic acid sensitive), which transfers reducing equivalents from glycolytic NADH to oxygen via glycerol-3-phosphate dehydrogenase and the ubiquinone pool [39,42–44]. *P*. *serpens* NADH-ubiquinone oxidoreductases were previously characterized [45], and also identified in this work (S1 Table).

As previously mentioned, *T*. *cruzi* is the human pathogen with the largest number of orthologous proteins with *P serpens*. Given this, *P*. *serpens*–a phytoparasite harmless to humans–has been attracting medical interest due its immunological cross-reactivity with *T*. *cruzi*, the etiological agent of Chagas disease [24,46,47]. The immunological cross-reactivity between *P*. *serpens* and *T*. *cruzi* has been well described, as the two species share common antigens. BALB/c mice immunization by intraperitoneal or oral route with *P*. *serpens* induces protective immunity against *T*. *cruzi* infection [24]. Moreover, the protective immunity provided by oral immunization is associated with enhanced NO production during the acute phase of *T*. *cruzi* infection [23] Also, immunization is able to attenuate thrombocytopenia and leukopenia during acute infection in mice [47]. In contrast, antibodies present in the sera of humans affected by Chagas disease react with 22 different *P*. *serpens* antigens [22], supporting the immunological cross reacting between both species.

The present *P*. *serpens* proteome analysis identified a new range of proteins, which may contribute to the process of immunological cross-reactivity. Among these proteins, some have already been described as candidates for potential vaccines against infections caused by kinetoplastids, such as the KMP-11 (kinetoplastid membrane protein-11) protein family [48,49].

The development of an efficient human vaccine against *T*. *cruzi* infection has been prevented by difficulties such as controversy about its genetic complexity and a limited set of engineering techniques for genome manipulation. Therefore, the development of a prophylactic vaccine able to reduce the parasite burden in humans and its reservoirs has become a challenge [50]. As mentioned before, *P*. *serpens* has potential for the development of vaccines against *T*. *cruzi* infection [22–24,46]. As an intracellular parasite, *T*. *cruzi* promotes the presentation of MHC class I epitopes by mammalian infected cells. [51]. A proteomic and immunoinformatics analysis using trypomastigote forms of *T*. *cruzi* predicted a total of 296 proteins as being able to produce major histocompatibility complex (MHC) class I epitopes [52]. Here, we showed that fourteen of those predicted proteins are found in the *P*. *serpens* proteome and, consequently, could perhaps originate the same MHC I epitopes (Table 1). Therefore, our results revealed proteins that could support the immunological cross-reactivity between *P*. *serpens* and *T*. *cruzi*. The possible use of these proteins in the development of a *P*. *serpens*-based safe vaccine should result in considerable advancement in the treatment of Chagas disease.

**Table 1:** *P*. *serpens* proteins that could potentially generate MHC I epitopes against *T*. *cruzi* trypomastigotes.

Cell fraction	Accession number	-10logP	Coverage (%)	#Peptides	#Unique	Avg. Mass	Description
**TOTAL**	Q8STF3|Q8STF3_TRYCR	306.15	69	51	1	49700	Beta tubulin 1.9
**TOTAL**	Q01530|Q01530_TRYCR	213.92	19	16	1	69548	Major paraflagellar rod protein
**CYT**	Q4DZ98|Q4DZ98_TRYCC	219.27	19	15	2	46444	Enolase putative
**CYT**	Q4CLA1|Q4CLA1_TRYCC	334.24	64	49	1	49799	Alpha tubulin putative
**TOTAL**	Q4CLA1|Q4CLA1_TRYCC	330.48	66	47	2	49799	Alpha tubulin putative
**HDO**	Q4DZ41|RS3A2_TRYCC	76.03	13	2	1	29855	40S ribosomal protein S3a-2
**CYT**	Q4DYK2|Q4DYK2_TRYCC	167.07	13	7	2	34955	ADP ATP carrier protein 1 mitochondrial putative
**TOTAL**	Q4DYK2|Q4DYK2_TRYCC	151.00	16	6	1	34955	ADP ATP carrier protein 1 mitochondrial putative
**HDO**	Q4D3P5|Q4D3P5_TRYCC	71.81	7	4	1	52144	Hexokinase
**CYT**	I6LE92|I6LE92_TRYCR	83.31	8	2	1	41969	Actin
**TOTAL**	I6LE92|I6LE92_TRYCR	122.33	18	4	1	41969	Actin
**TOTAL**	Q4CVR9|Q4CVR9_TRYCC	206.32	21	13	2	70990	Heat shock 70 kDa protein mitochondrial putative
**HDO**	Q4D7Y8|Q4D7Y8_TRYCC	86.01	14	2	2	20677	ADP-ribosylation factor 1 putative
**HDO**	Q4D5K2|Q4D5K2_TRYCC	190.46	40	10	3	28375	60S ribosomal protein L2 putative
**CYT**	Q4D1S0|Q4D1S0_TRYCC	146.21	15	4	1	55554	Vacuolar ATP synthase subunit B putative
**HDO**	Q4CUL0|Q4CUL0_TRYCC	68.55	6	1	1	24144	40S ribosomal protein S3 putative
**TOTAL**	Q4CUL0|Q4CUL0_TRYCC	128.75	21	5	1	24144	40S ribosomal protein S3 putative
**CYT**	Q4DWG6|Q4DWG6_TRYCC	79.75	4	2	1	59186	Chaperonin containing T-complex protein putative

Furthermore, the present homology-based *P*. *serpens* proteome analyses generated information relating to biological features, including its’ metabolism, and should contribute to the annotation and assembly of the genome [53]. In addition, this work may lead to a better understanding of the biology, biochemistry and evolutionary history of *P*. *serpens*.

## Supporting information

S1 Table Proteins identified in each *P*. *serpens* fraction (CYT, HDO and TOTAL). (XLSX)

S2 Table Peptides identified in the *P*. *serpens* fractions. (XLSX)

S1 Fig Alignment of phosphoglycerate mutase (A), citrate lyase (B) and glucosamine-6-phosphate deaminase (C) protein sequences from. *Phytomonas* sp HART1 with the non-annotated *P*. *serpens* genome sequence [9] using tblastn NCBI (http://blast.ncbi.nlm.nih.gov/Blast.cgi?PAGE_TYPE=BlastSearch&PROG_DEF=blastn&BLAST_SPEC=Assembly&ASSEMBLY_NAME=GCA_000331125.1.).
(TIF)

S1 Database Database uniprot-kinetoplastea. Local database comprising available Kinetoplastea parasite protein sequences for more than 200 different species (http://www.uniprot.org; release oct_2015) including two *Phytomonas* spp. (HART1 from group H and EM1 from group D), *Trypanosoma cruzi* (CL Brener), *Trypanosoma brucei* and *Leishmania infantum*.
(RAR)
