# Supplementary figures and images for: Proteome analysis of Phytomonas serpens, a phytoparasite of medical interest
Source: PLoS One. 2018 Oct 10;13(10):e0204818. doi: 10.1371/journal.pone.0204818 (PMC6179244; doi:10.1371/journal.pone.0204818)

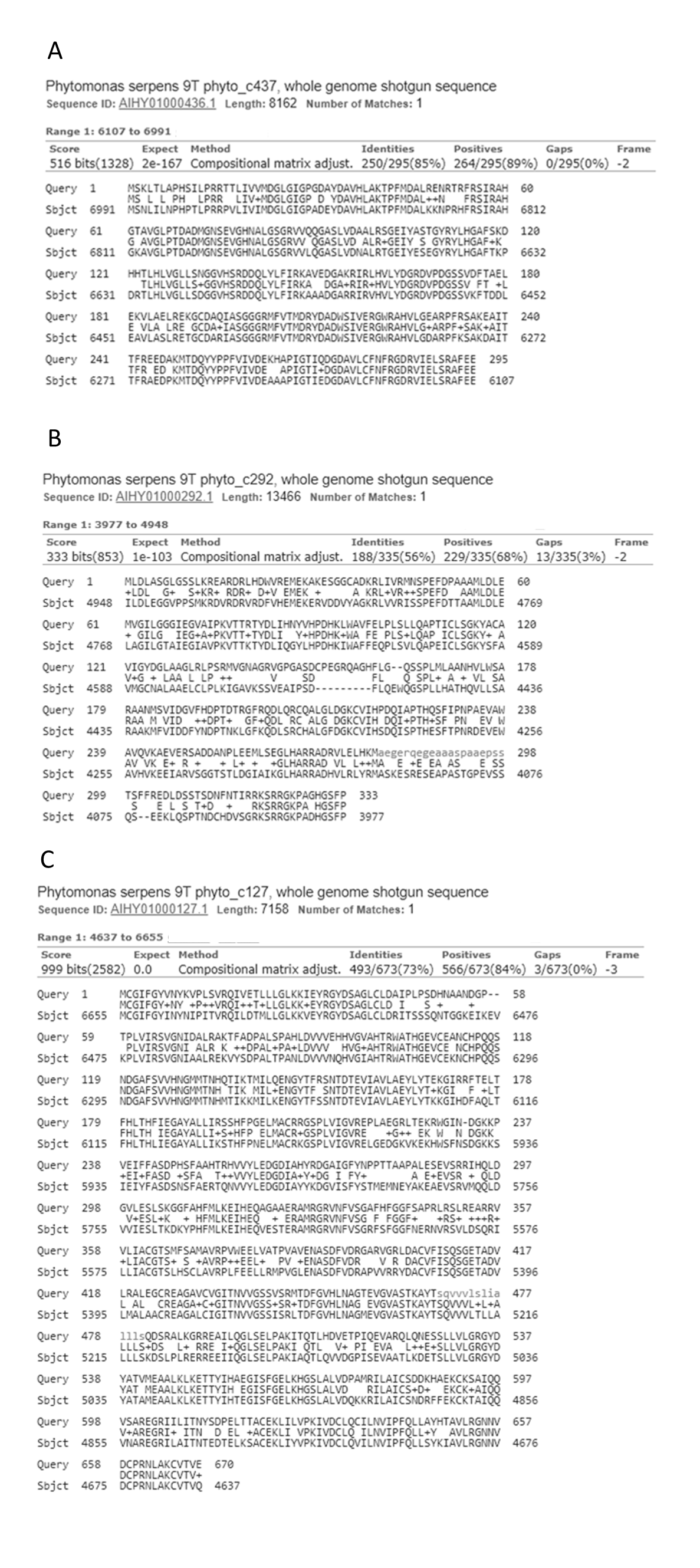

Supplement: S1 Fig — Phytomonas sp HART1 with the non-annotated P. serpens genome sequence [9] using tblastn NCBI (http://blast.ncbi.nlm.nih.gov/Blast.cgi?PAGE_TYPE=BlastSearch&PROG_DEF=blastn&BLAST_SPEC=Assembly&ASSEMBLY_NAME=GCA_000331125.1.). (TIF) [file pone.0204818.s003.tif]
